# Three-dimensional ultrastructural and histomorphological analysis of the periodontal ligament with occlusal hypofunction via focused ion beam/scanning electron microscope tomography

**DOI:** 10.1038/s41598-019-45963-w

**Published:** 2019-07-02

**Authors:** Shingo Hirashima, Keisuke Ohta, Tomonoshin Kanazawa, Akinobu Togo, Tatsuyuki Kakuma, Jingo Kusukawa, Kei-ichiro Nakamura

**Affiliations:** 1 Division of Microscopic and Developmental Anatomy, Department of Anatomy, Kurume University School of Medicine, Kurume, 830-0011 Japan; 2 Dental and Oral Medical Center, Kurume University School of Medicine, Kurume, 830-0011 Japan; 3 Advanced Imaging Research Center, Kurume University School of Medicine, Kurume, 830-0011 Japan; 4 Biostatics Center, Kurume University, Kurume, 830-0011 Japan

**Keywords:** Cellular imaging, Electron microscopy

## Abstract

The periodontal ligament (PDL) maintains the environment and function of the periodontium. The PDL has been remodelled in accordance with changes in mechanical loading. Three-dimensional (3D) structural data provide essential information regarding PDL function and dysfunction. However, changes in mechanical loading associated with structural changes in the PDL are poorly understood at the mesoscale. This study aimed to investigate 3D ultrastructural and histomorphometric changes in PDL cells and fibres associated with unloading condition (occlusal hypofunction), using focused ion beam/scanning electron microscope tomography, and to quantitatively analyse the structural properties of PDL cells and fibres. PDL cells formed cellular networks upon morphological changes induced via changes in mechanical loading condition. Drastic changes were observed in a horizontal array of cells, with a sparse and disorganised area of collagen bundles. Furthermore, collagen bundles tended to be thinner than those in the control group. FIB/SEM tomography enables easier acquisition of serial ultrastructural images and quantitative 3D data. This method is powerful for revealing 3D architecture in complex tissues. Our results may help elucidate architectural changes in the PDL microenvironment during changes in mechanical loading condition and regeneration, and advance a wide variety of treatments in dentistry.

## Introduction

The periodontal ligament (PDL) is a soft connective tissue comprising collagen fibre bundles and cells between the tooth cementum and the alveolar bone. The PDL essentially maintains the environment and function of the periodontium and serves as a structural anchor for teeth, facilitates proprioception, and maintains nutrition, homeostasis, and regeneration^1,2^. Therefore, PDL is a biologically and clinically significant tissue in dentistry^1,3,4^.

The PDL, as an anchoring structure, distributes externally applied force, e.g., occlusal loading or orthodontic forces, to the contiguous alveolar bone via Sharpey’s fibre. PDL has been previously remodelled through changes of mechanical loading condition^5^. Properties of the PDL and bone-associated changes in mechanical loading condition have been previously analysed *in vitro* and *in vivo* via a molecular genetics approach^1,5,6^. The PDL has a high turnover rate and can easily adapt to microenvironmental changes^1^. Furthermore, the effect of mechanical loading condition has been suggested to influence type I collagen expression in PDL cells^7^. Additionally, PDL cells express various osteoclastogenic molecules and play a significant dual role in remodelling alveolar bones^8^. The teeth are intermittently exposed to mechanical loading (occlusion). When this condition is changed (continuous or unloading), PDL and bone remodelling occurs, causing tooth movement. In clinical dentistry, tooth movements including tooth extrusion are often exploited for orthodontic, periodontal, and bone formation treatment. Extrusion of a tooth with loss of antagonist (opposing tooth) often occurs.

Proper comprehension of the targeted normal tissue is important for a more effective treatment. Data from three-dimensional (3D) structural analyses help determine structure-function and structure-dysfunction relationships. However, structural changes in the PDL associated with changes in mechanical loading are poorly understood on a mesoscale^9^ since such assessments are challenging when using only conventional methods.

Three-dimensional (3D) imaging methods have been recently developed including 3D direct visualisation via high-resolution micro-CT^10,11^, deep multi-photon imaging^12^, serial block face imaging, and scanning electron microscopy (SEM)-based quantitative analysis^13^. Novel 3D methods may provide novel insights into various tissues. Hence, newer 3D imaging analyses of PDL structure on a mesoscale may help elucidate changes in the 3D architecture associated with changes in mechanical loading.

Focused ion beam-SEM (FIB/SEM) tomography has recently been developed as a novel analytical method^14,15^, thus enabling higher-resolution imaging and quantitative analysis of structural properties. We previously reported the 3D ultrastructure of organelles^15,16^ and tissues^17,18^, including normal PDL cells^19^ and fibres^20^ via FIB/SEM tomography.

In this study, we investigated 3D ultrastructural and histomorphometric changes in PDL cells and fibres associated with changes in mechanical loading condition (from loading to unloading; occlusal hypofunction) via FIB/SEM tomography.

## Results

### Single section analysis using FIB/SEM (block face imaging)

**Figure 1:**
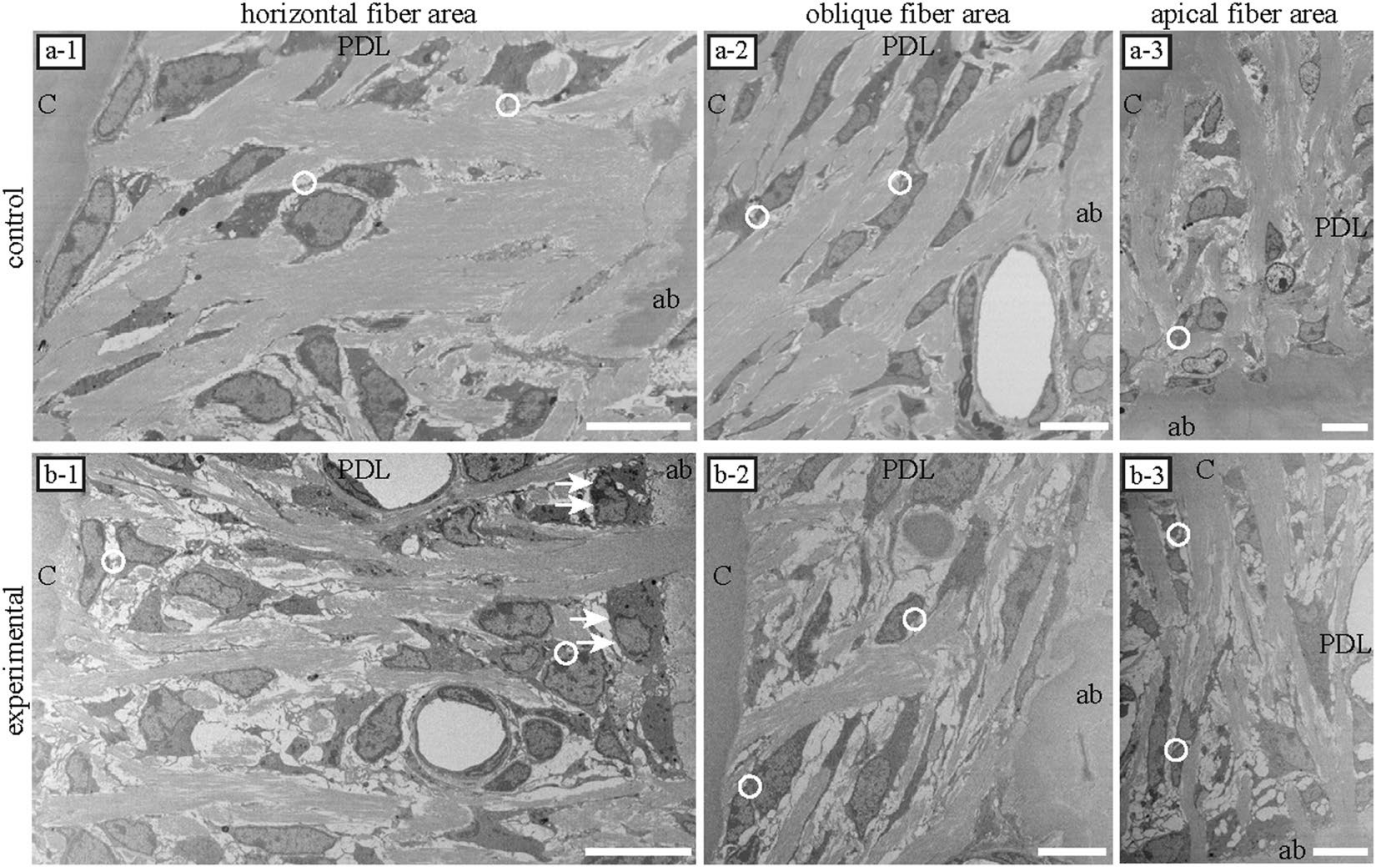
Single sections imaged via electron microscopy (EM). In both groups, periodontal ligament cells were spindle-shaped and parallelly oriented to the collagen bundle. Cellular processes were observed among the PDL fibres. Cells interacted with adjacent cells (white circle). The area of cellular interaction, which was in contact with other surrounding cells, was narrow. **(a-1~3)** Collagen bundles tended to be dense in each area of the control group. **(b-1~3)** Collagen bundles were disorganised in each area of the experimental group. In the area displaying horizontal fibres in the experimental group, osteoblast-like cells were observed on the surface of the alveolar bone (arrows). ab, alveolar bone; PDL, periodontal ligament; c, cementum. Scale bars: 10 μm for all panels.

In both the control and experimental groups, PDL cells were spindle-shaped and displayed a parallel orientation to the PDL collagen fibres. Their processes segregated each collagen bundle. Cells were in contact with adjacent cells. The area of direct intercellular contact was narrow (Fig. 1).

The cell surface was smooth in the control group (Fig. 1a-1~3). In contrast, small cellular processes were significantly more numerous in the experimental group than in the control group (Fig. 1b-1~3).

In the area of horizontal fibres in the experimental group, osteoblast-like cells were observed on the surface of alveolar bones (Fig. 1b-1; arrows).

Collagen bundles were sparse and disorganised in each area of the experimental group (Fig. 1b-1~3), although collagen bundles tended to be dense in each area of the control group (Fig. 1a-1~3).

### FIB/SEM tomography: serial cross-sections

**Figure 2:**
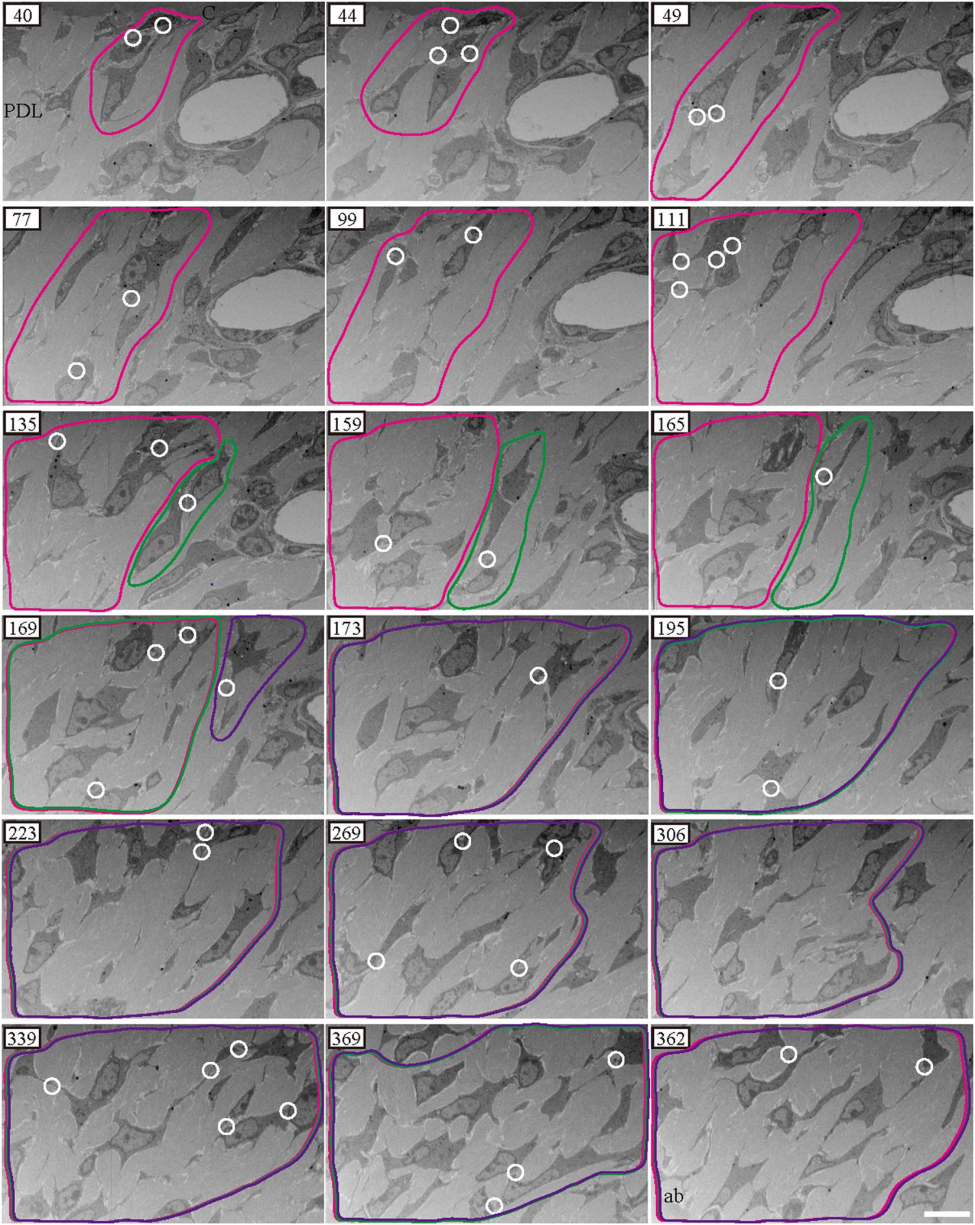
Serial cross-sections imaged via focused ion beam/scanning electron microscopy tomography for the control group in horizontal fibre area. White circles and ellipses represent intercellular interactions. Magenta, green, and purple regions represent regions of cell contact with other cells. In serial cross-sections, the region of intercellular contact was large, extending from the alveolar bone to the cementum. Magenta and green regions in slices 40–165 and 169 are separate areas. Two areas in slices 169 and 173 overlapped, and they have been magnified. ab, alveolar bone; PDL, periodontal ligament; c, cementum. Scale bars: 10 μm.

**Figure 3:**
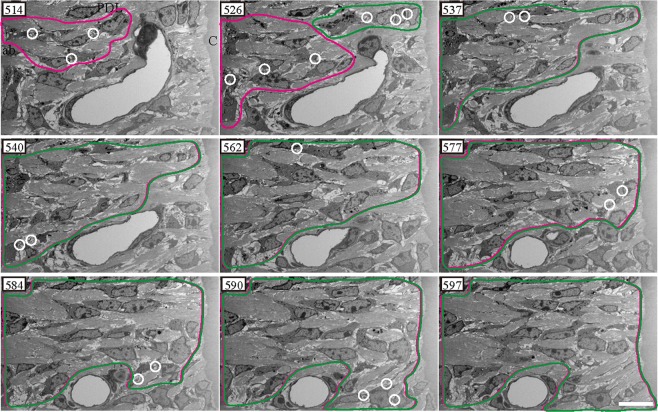
Serial cross-sections imaged using focused ion beam/scanning electron microscopy tomography for the experimental group in horizontal fibre area. White circles and ellipses represent regions of intercellular contact. Magenta and green regions represent regions of cell contact with other cells. In serial cross-sections, regions of the cells in direct contact with surrounding cells overlapped. The region of intercellular contact was large, ranging from the bone to the cementum. Magenta and green regions in slices 514–526 are separate areas. The two areas in slice 537 are overlapping and the overlapping region is expanded. ab, alveolar bone; PDL, periodontal ligament; c, cementum. Scale bars: 10 μm.

In both the control and experimental groups, PDL cells interacted with one another (Figs 2 and 3). In serial cross-sections, the intercellular contact region of PDL cells expanded and overlapped at different intercellular contact regions when the number of serial cross sections was increased (Figs 2 and 3; magenta, green, and purple). Finally, the area of intercellular interactions was large, spanning from the bone to the cementum. Thus, in single sections, cells were apparently not in contact. PDL cells appeared to be in contact with only surrounding cells; however, in serial cross-sections, cells, including distant cells, displayed indirect contact, by contacting the surrounding PDL cells. This widespread intercellular contact was observed in each observation area (Supplementary Fig. S1~S4)

Collagen bundles were sparse and disorganised throughout the experimental group (Fig. 3), although they tended to be dense in each area in the control group (Fig. 2) as observed during single section analysis.

### FIB/SEM tomography: 3D-structure reconstruction of PDL cells

We reconstructed the 3D structure of PDL cells and fibres from the data of FIB/SEM tomography.

**Figure 4:**
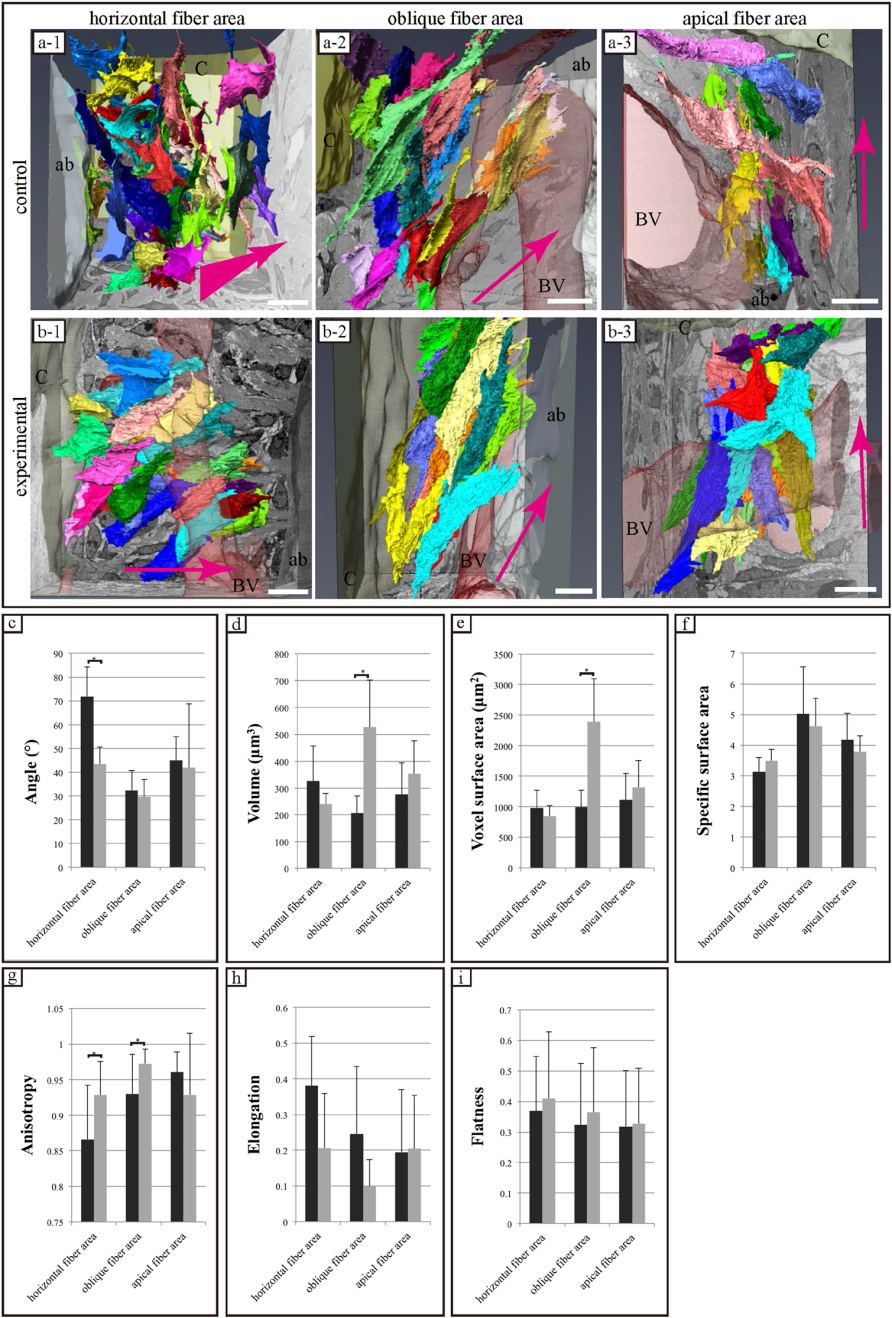
Comparisons of the three-dimensional structure and structural properties of periodontal ligament (PDL) cells in both groups. (**a-1~3**,**b-1~3**) Reconstructed 3D images of PDL cells. The direction of PDL fibres is shown by the magenta arrows. The arrays of cells and PDL fibre bundles differ in the observed area in each group. ab, alveolar bone; c cementum; BV, blood vessel. Scale bar: 10 μm for all panels. (**c~i**) Quantitative analysis of cell morphology in each area of both groups was performed via focused ion beam/scanning electron microscopy tomography. The angle between the long axis of each cell and the PDL fibre bundle is shown. The histogram shows the mean values for each area in both groups and the error bars represent standard deviations. The asterisk indicates a significant difference (P < 0.05).

In both the control and experimental groups, cells displayed a flattened morphology with long processes and did not have a spindle-like shape. PDL cells interacted with adjacent cells and formed a widespread cellular network between the cementum and bone (Fig. 4a-1~3,b-1~3).

The orientation of cells along PDL collagen fibres differed in different areas observed in both groups. In horizontal fibres, PDL cells in the control group tended to be oriented along the longitudinal axis at vertical angles to the direction of the fibre bundles (Fig. 4a-1). Conversely, cells in the experimental group were parallelly oriented with collagen bundles at the same site (Fig. 4b-1). In areas of oblique fibres, PDL cells in both groups were parallelly oriented with collagen bundles (Fig. 4a-2,b-2). In the apical fibre area, PDL cells exhibited a random orientation relative to the PDL fibres in both groups (Fig. 4a-3,b-3). In areas displaying horizontal, oblique, and apical fibres, the average angles of PDL cells in the control group were respectively 71.89 ± 12.50, 32.34 ± 8.44, and 45.03 ± 9.81°. In areas displaying horizontal, oblique, and apical fibres, the average angles of PDL cells in the experimental group were respectively 43.34 ± 7.24, 29.55 ± 7.29, and 41.81 ± 26.89°. The PDL cellular angle in the area displaying horizontal fibres was significantly different between the control and experimental groups (P < 0.05). No differences were observed in the angle of the PDL cells in areas displaying oblique and apical fibres between the control and experimental groups (Fig. 4c).

In areas displaying horizontal, oblique, and apical fibres, the average volumes of PDL cells in the control and experimental groups were 326.11 ± 130.98, 207.89 ± 62.30, and 276.71 ± 117.60 μm^3^ and 241.68 ± 38.28, 526.59 ± 175.65, and 353.66 ± 122.93 μm^3^; average voxel surface areas in the control and experimental groups were 982.41 ± 286.25, 994.68 ± 280.07, and 1114.06 ± 435.27 μm^2^ and 845.24 ± 170.09, 2387.99 ± 702.29, and 1318.95 ± 436.24 μm^2^; average specific surface areas in the control and experimental groups were 3.13 ± 0.45, 5.01 ± 1.54, and 4.16 ± 0.86 μm^2^ and 3.49 ± 0.36, 4.62 ± 0.91, and 3.78 ± 0.52 μm^2^; average anisotropies in the control and experimental groups were 0.86 ± 0.07, 0.93 ± 0.05, and 0.96 ± 0.02 and 0.92 ± 0.04, 0.97 ± 0.02, and 0.92 ± 0.08; average elongation ratios in the control and experimental groups were 0.38 ± 0.13, 0.24 ± 0.18, and 0.19 ± 0.17 and 0.20 ± 0.15, 0.09 ± 0.07, and 0.20 ± 0.15; and average flatness in the control and experimental groups were 0.36 ± 0.17, 0.32 ± 0.20, and 0.31 ± 0.18 µm^2^ and 0.41 ± 0.21, 0.36 ± 0.21, and 0.32 ± 0.17 μm^2^, respectively.

In areas displaying oblique fibres, average cell volume and voxel surface area differed significantly between the control and experimental groups (P < 0.05), with no differences between areas displaying horizontal and apical fibres (Fig. 4d,e). Specific surface area, average elongation ratio, and average flatness did not differ significantly among all areas (Fig. 4f,h,i). Average anisotropy in the control group differed significantly between the areas displaying horizontal and oblique fibres from those in the experimental group (P < 0.05), but not in areas displaying apical fibres (Fig. 4g).

### FIB/SEM tomography: 3D-structure reconstruction of PDL fibres

**Figure 5:**
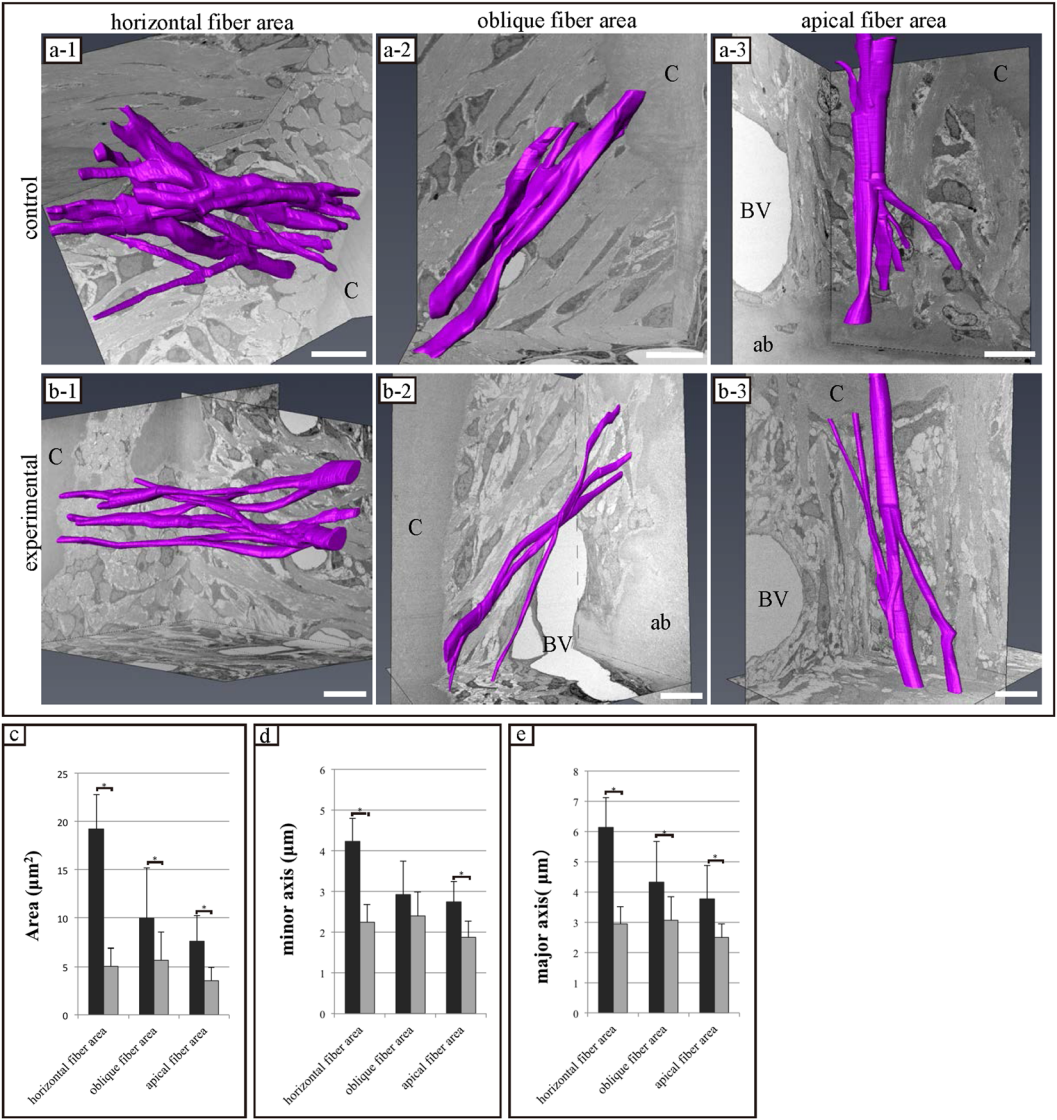
Comparison of three-dimensional structure and structural properties of periodontal ligament (PDL) fibres (collagen bundle) in each area of both groups. (**a-1~3**,**b-1~3**) Reconstructed 3D images of PDL bundles. Collagen bundles displaying similar morphology in both groups. The structure of the collagen bundle in the area displaying horizontal fibres is an extensive meshwork containing numerous interconnecting bundles and branches. The structures of the collagen bundles in areas displaying oblique and apical fibres are chain-like structures containing a few bundles interconnected with branches. ab, alveolar bone; c, cementum; BV, blood vessel. Scale bar: 10 μm for all panels. (**c**~**e**) Results of quantitative analysis of collagen bundle morphology as assessed via focused ion beam/scanning electron microscopy tomography. The histogram shows the means for each group and the error bars represent standard deviations. The asterisk indicates a significant difference (P < 0.05).

In both groups, PDL fibres displayed an extensively branched structure. Furthermore, the structure of collagen bundles differed among the areas observed. PDL fibres in areas displaying horizontal fibres were in an extensive meshwork containing multiple interconnected and branched bundles of fibres (Fig. 5a-1,b-1). In contrast, the structure of PDL fibres in areas displaying oblique and apical fibres was chain-like, containing a few interconnected or branched bundles (Fig. 5a-2 and 3,b-2 and 3). Although the structure of PDL fibres in each area was similar in both groups, PDL fibres tended to be finer in the experimental group than in the control group.

We quantitatively analysed the cross-sectional area, and the lengths of the minor and major axes from 3D images of the collagen bundles. The cross-sectional areas of the collagen bundles in areas displaying horizontal, oblique, and apical fibres in the control and experimental groups were 19.20 ± 3.57, 9.98 ± 5.20, and 7.60 ± 2.65 µm^2^ and 5.03 ± 1.87, 5.67 ± 2.84, and 3.56 ± 1.34 µm^2^, respectively, differing significantly between the control and experimental groups in each area (P < 0.05) (Fig. 5c).

The minor axis values of cross-sections of collagen bundles in areas displaying horizontal, oblique, and apical fibres in the control and experimental groups were 4.23 ± 0.55, 2.92 ± 0.81, and 2.74 ± 0.50 µm and 2.24 ± 0.43, 2.39 ± 0.59, and 1.87 ± 0.39 µm, respectively. Values for the horizontal and apical fibres differed significantly between the control and experimental groups (P < 0.05), with no significant difference in the minor axis value in areas displaying oblique fibres (Fig. 5d).

The major axis values of cross-sections of collagen bundles in areas displaying horizontal, oblique, and apical fibres in the control and experimental groups were 6.13 ± 0.98, 4.32 ± 1.34, and 3.76 ± 1.11 µm and 2.95 ± 0.55, 3.06 ± 0.78, and 2.48 ± 0.45 µm, respectively, differing significantly between the control and experimental groups in each area (P < 0.05) (Fig. 5e).

## Discussion

In this study, we clarified firstly and proved directly the 3D ultrastructural changes in PDL cells associated with changes in mechanical loading condition (from loading to unloading) via FIB/SEM tomography. The present results indicate that PDL cells established cellular networks, accompanied with morphological changes with changes in the mechanical loading condition. Collagen bundles in the experimental group became thinner than those in the control group. FIB/SEM tomography is useful for 3D ultrastructural and histomorphological analysis of non-uniform tissue (e.g. a complex of soft and hard tissue), although it is difficult to perform when using only conventional methods. This method enables comprehensive visualisation of samples with a size of more than 1 mm via block face imaging, although the size of samples for electron microscope is usually less than or equal to 1 mm. After comprehensive visualisation, the area for FIB/SEM imaging was selected (limited to 100 × 100 × 100 μm^3^) from the complex tissue. The method for specimen preparation for FIB/SEM as used in this study is less destructive and susceptible to artefacts than the conventional methods, although complex tissues with different hardness are usually susceptible to artefacts. FIB/SEM tomography is a suitable and a powerful method for comprehending the architecture of complex tissues including the bone-PDL-tooth complex.

In the control and experimental groups in the present study, PDL cells interacted with adjacent cells and established an extensive cellular network between the cementum and the alveolar bone, although the type of junction could not be determined in detail. Cellular networks have been reported in other tissues. These cellular networks may have formed via gap junctions related to various cell-cell interactions, cellular activities, and tissue functions. Furthermore, astrocytoma cells reportedly display chemotherapeutic resistance via a cellular network^21^. Moreover, PDL cells interact via gap or adherens junctions^1,22^. In addition, PDL cells including fibroblasts may contribute to osteoclastogenesis^8^ and partially regulate osteoblast behaviour via gap junctions^23^. We previously demonstrated that PDL cells, osteoblast-like cells, and osteocyte-like cells contacted each other^19^. Applied mechanical force further accelerates alveolar bone remodelling and PDL fibre bundles^1,5^. Furthermore, the width of the PDL was kept constant during the regeneration process^2^. The present results suggest that extensive cellular networks via gap junctions may form the structural basis for bone remodelling and modifications in fibres accompanied with changes in mechanical loading condition. In the future, we intend to clarify the type of cellular junction and the extent of cellular interactions (cellular network only in the PDL or the cellular network over PDL-alveolar bones).

Furthermore, the angle formed by the long axis of the cell and the collagen bundle was altered with changes of mechanical loading condition in only the area displaying horizontal fibres. Additionally, previous studies have reported^24^ the onset of collagen fibre alignment, especially at the attached ends of cellular pseudopods and between adjacent cells attached to the same collagen bundles. Thereafter, cellular morphology was altered with spreading and bipolar elongation of the cellular processes along the aligned collagen fibres. Consequently, in areas displaying horizontal fibres, cellular orientation may change due to the extrusion of tooth caused by occlusal hypofunction, which could result in PDL alteration.

The strain distribution in the PDL is suggested to be non-uniform upon finite element analysis^25^. Moreover, the effect of mechanical loading in the vertical direction on the PDL around the cervical area of the tooth is reportedly extremely small and that in the horizontal direction on the PDL around the cervical area of the tooth is larger. Moreover, changes in the area of horizontal fibres are larger than those in other areas. Notwithstanding the difference in the angle between the oblique and apical areas, both areas are constantly susceptible to changes in mechanical loading in either direction of loading. Therefore, no significant difference would be expected in the change in the angle between both areas in the control and experimental groups.

Furthermore, other parameters for analysis, including volume, voxel surface area, and anisotropy, can be analysed via non-uniform distribution of strain. Notwithstanding changes in mechanical loading, we observed no significant difference in specific surface area, elongation ratio, and flatness for each observed area. Higher values of specific surface area are significant for applications including filtrations or catalysis^26^. There are reports that fibroblasts not only synthesise collagen but also degrade it on cell surfaces during remodelling and collagen turnover^27,28^. Collagen synthesis reportedly comprises intra-cellular and extra-cellular processes^7^. Furthermore, mechanical loading-induced type I collagen expression in PDL cells (fibroblasts) was magnitude-, duration-, and mode-dependent. It is reported that the expression of the type I collagen gene also responds (increasing or decreasing) to changes in mechanical loading condition^7^. Hence, when PDL fibres are remodelled at large distances between the tooth and the bone, a constant specific surface area of PDL cells that is unaffected by changes in mechanical loading is important.

The present results indicate that collagen bundles tend to become thinner with changes in mechanical loading condition in each area, although their 3D ultrastructure (mesh work or chain-like structure) remained unchanged. Orthodontic tooth movement reportedly induces changes in the morphology and mineralisation patterns of Sharpey’s fibres, thereby potentially affecting the mechanical strength of the periodontium^29^. In single section analysis of this study, collagen bundles in each observation area were sparse and disorganised, concurrent with previous reports^30,31^. Upon 3D analysis, not all collagen bundles were ruptured, despite being sparse and disorganised. Furthermore, in the present study, serial-slice SEM analysis revealed finer collagen bundles in the experimental group than in the control group. As collagen bundles became thinner, the PDL area was sparse upon single-section analysis, thereby potentially suggesting that collagen bundles became thinner upon remodelling and turnover of the bundles with changes in mechanical loading. Furthermore, in a dynamically stable situation, collagen bundles are expected to become thicker, organised, and dense.

The present study had several limitations. Firstly, the area of observation was limited (100 × 100 × 100 μm^3^). We performed mesoscale analysis of a part of the PDL. To elucidate changes in the PDL with changes in mechanical loading condition, the ultrastructure and histomorphometric characteristics of the whole PDL need to be analysed on a multi-scale together with other imaging methods. Secondly, we only used the rO-T-O staining method for FIB/SEM imaging. Morphological and quantitative changes in PDL cells and fibres with occlusal hypofunction were elucidated in this study. Future studies should focus on functional analyses, including 3D-structural analysis combined with immunohistochemistry. Finally, we used a semi-automated and manual procedure for 3D reconstruction of cells and collagen bundles. Although we used an appropriate procedure for each subject in this study, a lot of effort was required for the analysis. It is necessary to develop a simpler procedure using artificial intelligence in the future. Future studies are required to consider and perform functional analyses including 3D analysis combined with immunohistochemistry. However, we believe that the present results will benefit future studies in this field.

In conclusion, the present study describes the 3D ultrastructural and histomorphological changes in the PDL associated with changes in mechanical loading condition (occlusal hypofunction), as revealed via FIB/SEM tomography. Our results indicate that the angle between the fibre and the cells was altered while maintaining cellular interactions with changes in mechanical loading condition. In addition, the collagen bundle of the PDL became finer than that of the control group. Collagen bundles were sparse and disorganised. These novel findings further the current understanding of structural changes in the PDL with changes in mechanical loading on a mesoscale. Furthermore, the present results may help elucidate microenvironmental changes during changes in mechanical loading condition and regeneration, and advance a wide variety of treatments in dentistry.

## Materials and Methods

### Animals and experimental design

**Figure 6:**
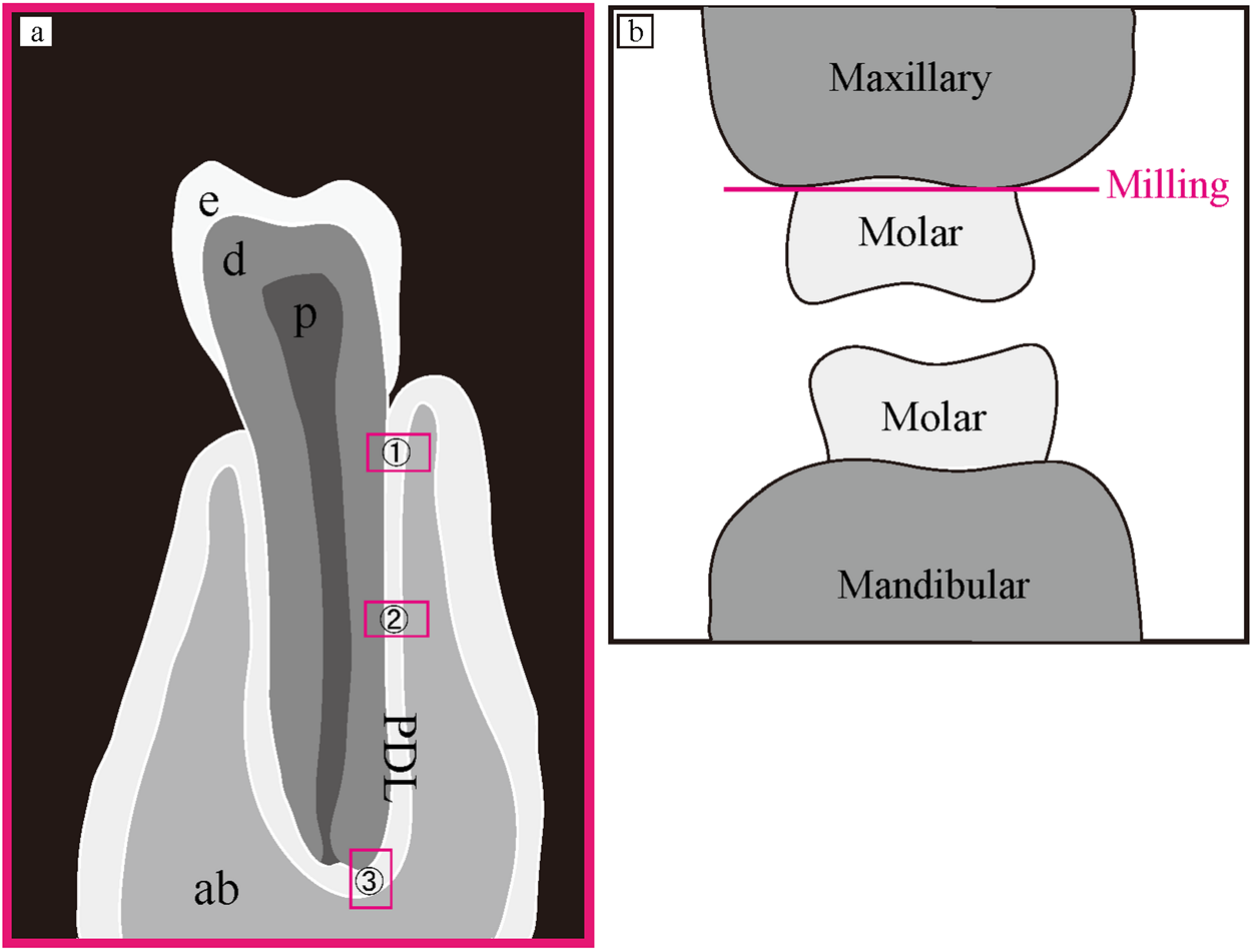
A schema of the observation area and surgery. (**a**) A schema of the observation area. We observed PDL in the (1) horizontal fibre area, (2) oblique fibre area, and (3) apical fibre area. ab, alveolar bone; PDL, periodontal ligament; e, enamel; d, dentin; c, cementum; P, dental pulp. (**b**) A schematic representation of the generation of the mouse model of occlusal hypofunction. Left maxillary molars were milled below their gingival margins using a dental steel bar.

All experiments were performed in accordance with the National Institutes of Health Guidelines for animal research. All animal procedures were approved by the Board for Animal Experiments of Kurume University. Six adult C57BL/6 mice were used in this study: three were used to generate a complete occlusal hypofunction model, and three were used as normal controls. Left maxillary molars were milled to prevent occlusion of the left mandibular molar. One week after surgery, three-dimensional histomorphological changes in PDL cells and collagen bundles of the first molar of the left mandible were observed and analysed via FIB/SEM tomography. The PDL region at the centre of the mesial root of the lower first molar was divided into three areas (Fig. 6a): (1) a horizontal fibre area, (2) an oblique fibre area, and (3) an apical fibre area. In total, 45 cells and 180 cross-sections of collagen bundles were analysed in each group (15 cells and 60 collagen bundles in each area). Tissue preparation, FIB/SEM tomography, and 3D reconstruction were performed as described in our previous studies^15,19,20,32^.

### Surgical generation of the mouse model of occlusal hypofunction

Mice were anaesthetised with isoflurane at a high flow rate of oxygen. Left maxillary molars were milled to prevent occlusion of the left mandibular molar below their gingival margins, using a dental steel bar (Fig. 6b). One week after surgery, animals were euthanised.

### Tissue preparation of EM specimens

Specimens were prepared as described previously^15,20,32^. Mice were anaesthetised with sodium pentobarbital (30 mg/kg) and transcardially perfused through the left ventricle with heparin (10 U/ml) in saline, followed by fixation with 2% paraformaldehyde and incubation in 2.5% glutaraldehyde in 0.1 M cacodylate (pH 7.3) buffer for EM. After perfusion, the mandible was dissected from the skull. The specimens were immersed in the same fixative for 2 h at 4 °C, washed in buffer, and then decalcified in Kalkitox solution (Wako Pure Chemical Industries, Ltd., Osaka, Japan) for 5 days. Thereafter, specimens were washed thrice in buffer for 10 min each.

Specimens were then diced and subjected to post-fixation processing and *en bloc* staining as described previously^15,19,20,32^. Briefly, after three washes in cacodylate buffer, specimens were postfixed for 2 h in a solution containing 2% osmium tetroxide and 1.5% potassium ferrocyanide in cacodylate buffer at 4 °C, washed thrice with distilled water (DW), and immersed in 1% thiocarbohydrazide solution for 1 h. After five washes with DW, the specimens were further immersed in 2% osmium tetroxide in DW and then washed thrice with DW. Specimens were then stained *en bloc* in a solution of 4% uranyl acetate dissolved in DW overnight for contrast enhancement and then washed with DW. Thereafter, the specimens were further stained with Walton’s lead aspartate solution for 2 h^33^, dehydrated in an ethanol series (25%, 50%, 70%, 80%, 90%, and twice in 100% ethanol for 5 min each), infiltrated with epoxy resin (Epon 812; TAAB, Aldermaston, UK), and polymerised for 72 h at 60 °C. The surfaces of the embedded specimens were exposed using a diamond knife on an Ultracut E microtome (Leica, Wetzlar, Germany). After trimming, the resin blocks were placed on a holder.

### FIB/SEM tomography and 3D reconstruction

FIB/SEM tomography analysis was performed as described previously^19,20^. Serial images of the block faces were acquired via repeated cycles of sample surface milling and imaging using Slice & View G2 operating software (FEI, Eindhoven, Netherlands). Milling was performed with a gallium ion beam at 30 kV with a current of 15 nA. The milling pitch was set to 100 nm/step and 800 cycles. Images were acquired at a landing energy of 2.5 keV. Additional acquisition parameters were as follows: beam current = 50.8 pA, dwell time = 6 µs/pixel, image size = 2048 × 1768 pixels, and pixel size = 36 nm/pixel. The resulting image stack, segmentation, and 3D reconstructed images were processed using Fiji software (http://fiji.sc/Fiji) and Amira 6.5 software (FEI Visualization Science Group, Burlington, MA, USA). Images were observed with optional X-Y-Z plane sectioning. The 3D morphology of the cells and collagen bundles was reconstructed using a semi-automated and manual procedure^17,20,32^.

From 3D reconstruction data, we performed quantitative analysis of whole cells and collagen bundles within the observation range. For each group, 45 cells were analysed including 15 cells in the horizontal fibre area, 15 cells in the oblique fibre area, and 15 cells in the apical fibre area (90 cells in total in both groups). The angle between each cell and collagen bundle was measured. Using Amira software and FIB/SEM tomography, we not only obtained the XY plane but also the XZ, YZ, and arbitrary planes in the observation range. We confirmed the plane in the fibre direction using the FIB/SEM tomography data. Additionally, planes containing the vertical aspect to the direction of a fibre were obtained. Each normal vector in the fibre bundle direction was then calculated. The normal vectors of the long axis of each cell were then calculated from 3D reconstruction data. To measure the angle of each cell relative to the direction of the fibre bundle, the inner product between the normal vectors of each cell and the direction of the fibre was calculated. In addition, the volume, voxel surface area, specific surface area, anisotropy, elongation, and flatness of PDL cells were calculated. Anisotropy was determined as 1 minus the ratio of the smallest to the largest eigenvalue, with spherical objects yielding small values close to 0. Elongation was determined as the ratio of the medium to the largest eigenvalue, with elongated objects yielding small values close to 0. Flatness was determined as the ratio of the smallest to the medium eigenvalue, with flat objects being assigned small values close to 0.

Thereafter, cross-sections of the collagen bundles within the observation range were quantitatively analysed. For each group, 180 cross-sections of collagen bundles were analysed, including 60 bundles in each of the three areas. The area and the minor and major axis lengths of cross-sections of the collagen bundles were measured (total of 360 cross-sections).

### Statistical analysis

The mixed effect model was used to evaluate differences between the control and experimental groups in each of the areas accounting for inter-correlation within a sample. For cells, the parameters for evaluation were as follows: angle between cells and the collagen bundles, volume, voxel surface area, specific surface area, anisotropy, elongation, flatness; collagen bundles: area and minor and major axis lengths of cross-sections of the collagen bundles. Results are presented as mean ± standard deviation (SD) values. Differences with P < 0.05 are considered statistically significant. Statistical analysis was performed using JMP version 13 (SAS Institute Inc., Cary, NC, USA).

## Supplementary information

Supplementary Figure S1~S4

## Data Availability

All data generated or analysed in this study are included in this article and the Supplementary Information Files.

## References

[1] Beertsen W, McCulloch CA, Sodek J (1997). The periodontal ligament: a unique, multifunctional connective tissue. Periodontol. 2000.

[2] Shimono M (2003). Regulatory mechanisms of periodontal regeneration. Microsc. Res. Tech..

[3] Nanci A, Bosshardt DD (2006). Structure of periodontal tissues in health and disease. Periodontol. 2000.

[4] Seo BM (2004). Investigation of multipotent postnatal stem cells from human periodontal ligament. Lancet.

[5] McCulloch CA, Lekic P, McKee MD (2000). Role of physical forces in regulating the form and function of the periodontal ligament. Periodontol. 2000.

[6] Lekic, P. & McCulloch, C. A. Periodontal ligament cell population: the central role of fibroblasts in creating a unique tissue. *Anat. Rec*. **245**, 327–341, 10.1002/(sici)1097-0185(199606)245:2<327::aid-ar15>3.0.co;2-r (1996). 10.1002/(SICI)1097-0185(199606)245:2<327::AID-AR15>3.0.CO;2-R 8769671

[7] Kaku M, Yamauchi M (2014). Mechano-regulation of collagen biosynthesis in periodontal ligament. J. Prosthodont. Res..

[8] Sokos D, Everts V, de Vries TJ (2015). Role of periodontal ligament fibroblasts in osteoclastogenesis: a review. J. Periodont. Res..

[9] Sear RP, Pagonabarraga I, Flaus A (2015). Life at the mesoscale: the self-organised cytoplasm and nucleoplasm. BMC Biophys..

[10] Naveh GR, Brumfeld V, Dean M, Shahar R, Weiner S (2014). Direct microCT imaging of non-mineralized connective tissues at high resolution. Connect. Tissue Res..

[11] Naveh GRS, Foster JE, Silva Santisteban TM, Yang X, Olsen BR (2018). Nonuniformity in ligaments is a structural strategy for optimizing functionality. Proc. Natl. Acad. Sci. USA.

[12] Acar M (2015). Deep imaging of bone marrow shows non-dividing stem cells are mainly perisinusoidal. Nature.

[13] Ohno N, Katoh M, Saitoh Y, Saitoh S, Ohno S (2015). Three-dimensional volume imaging with electron microscopy toward connectome. Microscopy (Oxf).

[14] Knott G, Marchman H, Wall D, Lich B (2008). Serial section scanning electron microscopy of adult brain tissue using focused ion beam milling. J. Neurosci..

[15] Ohta K (2012). Beam deceleration for block-face scanning electron microscopy of embedded biological tissue. Micron.

[16] Miyazono Y (2018). Uncoupled mitochondria quickly shorten along their long axis to form indented spheroids, instead of rings, in a fission-independent manner. Sci. Rep..

[17] Kanazawa T (2016). Histomorphometric and ultrastructural analysis of the tendon-bone interface after rotator cuff repair in a rat model. Sci. Rep..

[18] Yoshitomi M (2016). Three-dimensional ultrastructural analyses of anterior pituitary gland expose spatial relationships between endocrine cell secretory granule localization and capillary distribution. Sci. Rep..

[19] Hirashima S (2016). Three-dimensional ultrastructural analysis of cells in the periodontal ligament using focused ion beam/scanning electron microscope tomography. Sci. Rep..

[20] Hirashima S (2018). Three-dimensional ultrastructural analysis and histomorphometry of collagen bundles in the periodontal ligament using focused ion beam/scanning electron microscope tomography. J. Periodont. Res..

[21] Osswald M (2015). Brain tumour cells interconnect to a functional and resistant network. Nature.

[22] Beertsen W, Everts V (1980). Junctions between fibroblasts in mouse periodontal ligament. J. Periodont. Res..

[23] Pirraco RP, Cerqueira MT, Reis RL, Marques AP (2012). Fibroblasts regulate osteoblasts through gap junctional communication. Cytotherapy.

[24] Farsi JM, Aubin JE (1984). Microfilament rearrangements during fibroblast-induced contraction of three-dimensional hydrated collagen gels. Cell Motil..

[25] Qian L, Todo M, Morita Y, Matsushita Y, Koyano K (2009). Deformation analysis of the periodontium considering the viscoelasticity of the periodontal ligament. Dent. Mater..

[26] Katsogiannis KAG, Vladisavljevic GT, Georgiadou S, Rahmani R (2016). Assessing the increase in specific surface area for electrospun fibrous network due to pore induction. ACS Appl. Mater. Interfac..

[27] Cate AR, Deporter DA (1975). The degradative role of the fibroblast in the remodelling and turnover of collagen in soft connective tissue. Anat. Rec..

[28] ten Cate AR (1972). Morphological studies of fibrocytes in connective tissue undergoing rapid remodelling. J. Anat..

[29] Garant PR, Cho MI, Cullen MR (1982). Attachment of periodontal ligament fibroblasts to the extracellular matrix in the squirrel monkey. J. Periodont. Res..

[30] Levy GG, Mailland ML (1980). Histologic study of the effects of occlusal hypofunction following antagonist tooth extraction in the rat. J. Periodontol..

[31] Kaneko S, Ohashi K, Soma K, Yanagishita M (2001). Occlusal hypofunction causes changes of proteoglycan content in the rat periodontal ligament. J. Periodont. Res..

[32] Hirashima S (2015). Anchoring structure of the calvarial periosteum revealed by focused ion beam/scanning electron microscope tomography. Sci. Rep..

[33] Walton J (1979). Lead asparate, an en bloc contrast stain particularly useful for ultrastructural enzymology. J. Histochem. Cytochem..

