# Supplementary material for: Three-dimensional ultrastructural and histomorphological analysis of the periodontal ligament with occlusal hypofunction via focused ion beam/scanning electron microscope tomography
Source: Sci Rep. 2019 Jul 2;9:9520. doi: 10.1038/s41598-019-45963-w (PMC6606634; doi:10.1038/s41598-019-45963-w)
Supplement: Supplementary file 1 — Supplementary Figure S1~S4 [file 41598_2019_45963_MOESM1_ESM.pdf]

Three-dimensional ultrastructural and histomorphological analysis of the periodontal ligament with occlusal hypofunction via focused ion beam/scanning electron microscope tomography

Shingo Hirashima[*, 1, 2], Keisuke Ohta[1, 3], Tomonoshin Kanazawa[1], Akinobu Togo[3], Tatsuyuki Kakuma[4], Jingo Kusukawa[2], and Kei-ichiro Nakamura[1]

[1]Division of Microscopic and Developmental Anatomy, Department of Anatomy, Kurume University School of Medicine, Kurume 830-0011, Japan

[2]Dental and Oral Medical Center, Kurume University School of Medicine, Kurume 830-0011, Japan

[3]Advanced Imaging Research Center, Kurume University School of Medicine, Kurume 830-0011, Japan

[4] Biostatics Center, Kurume University, Kurume, 830-0011, Japan.

***Correspondence and requests for materials should be addressed to S. H. (e-mail: hirashima_shingo@med.kurume-u.ac.jp)

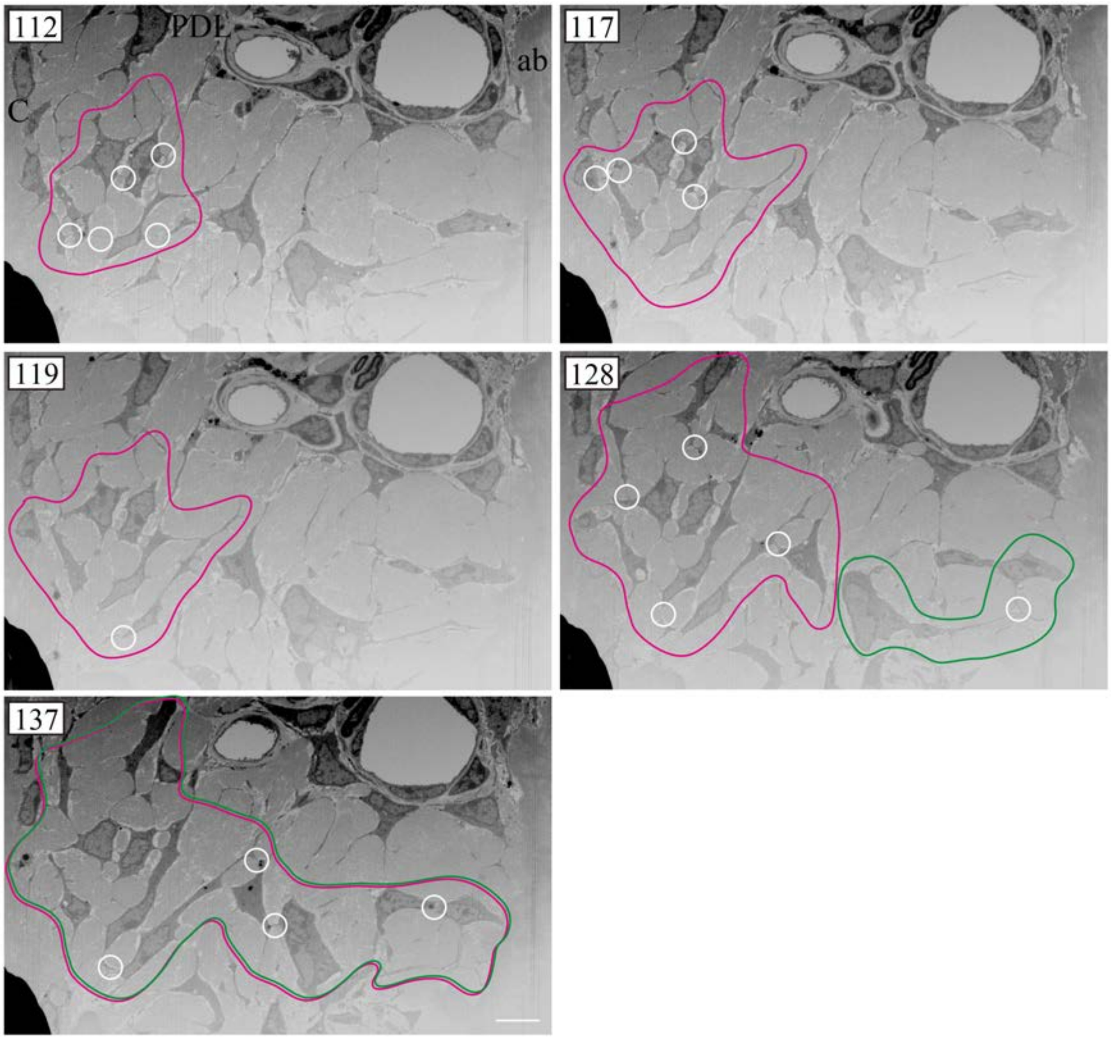

Supplementary Fig. S1 Serial cross-sections imaged via focused ion beam/scanning electron microscopy tomography for the control group in oblique fibre area of control group. White circles and ellipses represent intercellular interactions. Magenta and green regions represent regions of cell contact with other cells. In serial cross-sections, the region of intercellular contact was large, extending from the alveolar bone to the cementum. Magenta and green regions in slice 128 are separate areas. Two areas in slice 137 overlapped, and they have been magnified. ab, alveolar bone; PDL, periodontal ligament; c, cementum. Scale bars: 10 μm for all panels.

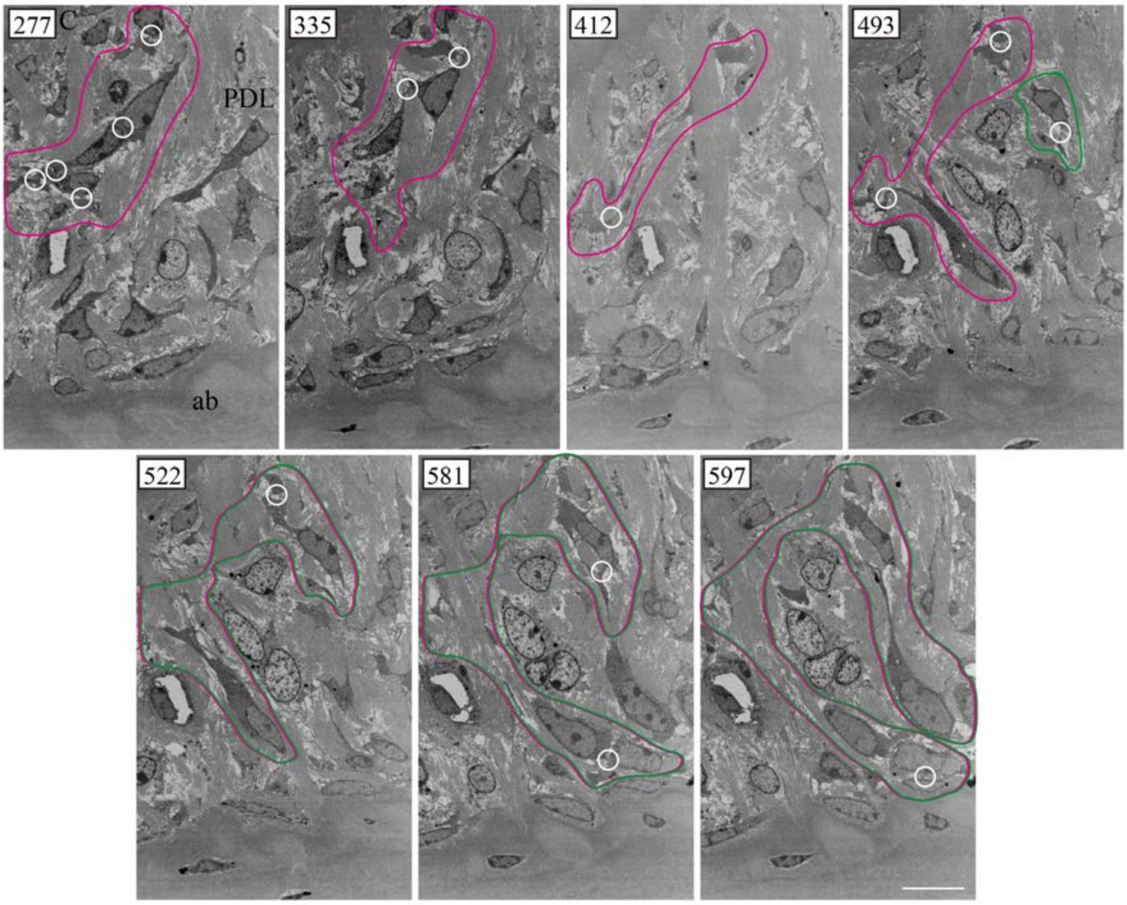

Supplementary Fig. S2 Serial cross-sections imaged via focused ion beam/scanning electron microscopy tomography for the control group in apical fibre area of control group. White circles and ellipses represent intercellular interactions. Magenta and green regions represent regions of cell contact with other cells. In serial cross-sections, the region of intercellular contact was large, extending from the alveolar bone to the cementum. Magenta and green regions in slice 493 are separate areas. Two areas in slice 522 overlapped, and they have been magnified. ab, alveolar bone; PDL, periodontal ligament; c, cementum. Scale bars: 10 μm for all panels.

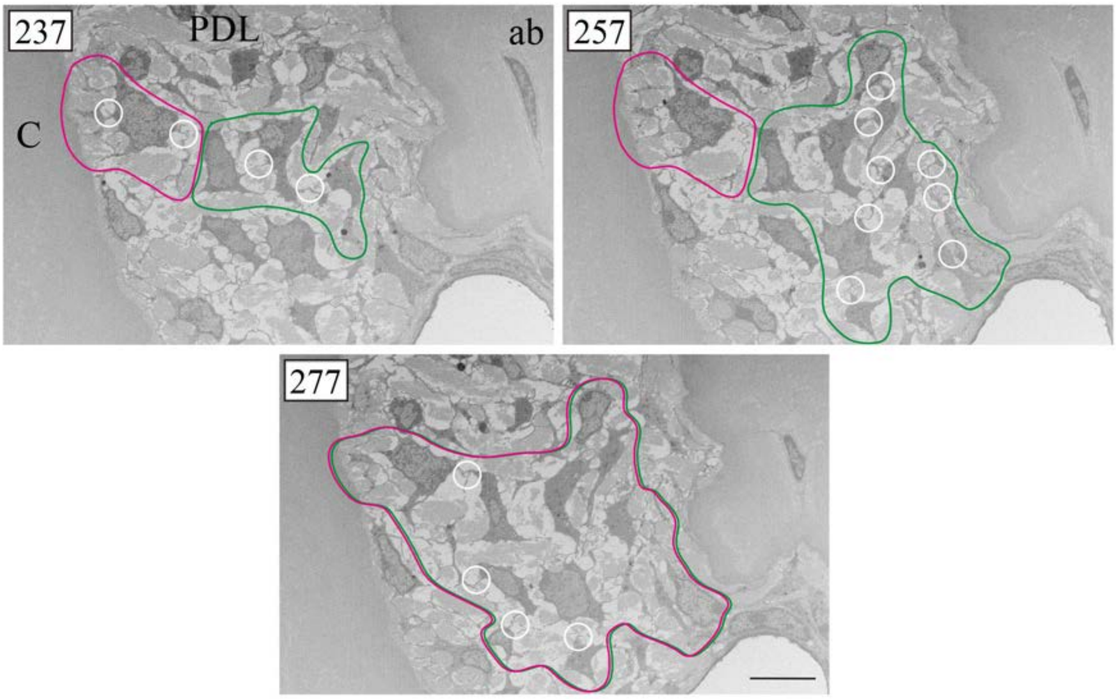

Supplementary Fig. S3 Serial cross-sections imaged via focused ion beam/scanning electron microscopy tomography for the experimental group in oblique fibre area of the experimental group. White circles and ellipses represent intercellular interactions. Magenta and green regions represent regions of cell contact with other cells. In serial cross-sections, the region of intercellular contact was large, extending from the alveolar bone to the cementum. Magenta and green regions in slices 237 and 257 are separate areas. Two areas in slice 277 overlapped, and they have been magnified. ab, alveolar bone; PDL, periodontal ligament; c, cementum. Scale bars: 10 µm for all panels.

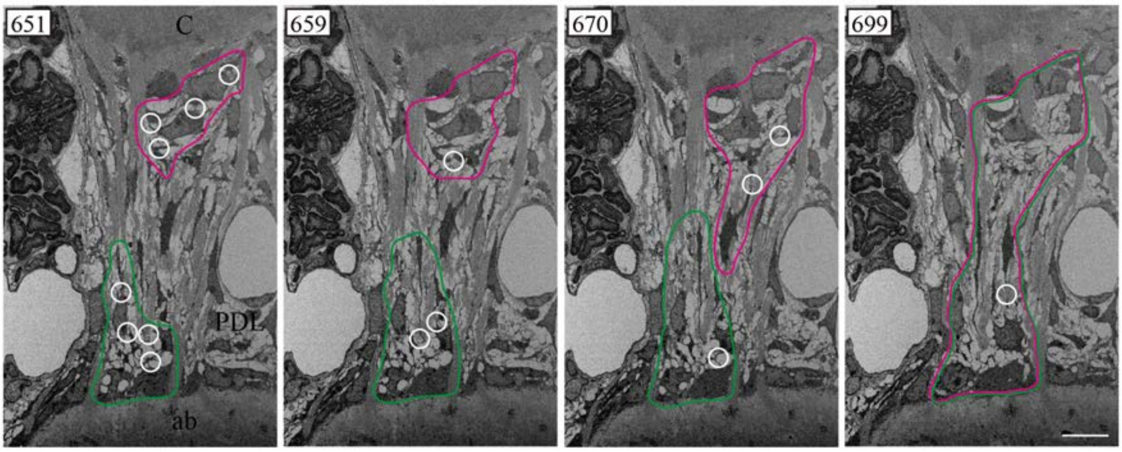

Supplementary Fig. S4 Serial cross-sections imaged via focused ion beam/scanning electron microscopy tomography for the experimental group in apical fibre area of the experimental group. White circles and ellipses represent intercellular interactions. Magenta and green regions represent regions of cell contact with other cells. In serial cross-sections, the region of intercellular contact was large, extending from the alveolar bone to the cementum. Magenta and green regions in slices 651, 659, and 670 are separate areas. Two areas in slice 699 overlapped, and they have been magnified. ab, alveolar bone; PDL, periodontal ligament; c, cementum. Scale bars: 10 μm for all panels.
